# Poultry Genomics Puts Meat on the Table

**DOI:** 10.1002/cfg.485

**Published:** 2005

**Authors:** Ed Smith, Olivier Pourquié, Dave Burt

**Affiliations:** 1 Department of Animal and Poultry Science Virginia Tech Blacksburg VA 24060 USA; 2 Stowers Institute for Medical Research 1000 East 50th Street Kansas City MO 64110 USA; 3 Department of Genomics and Genetics Roslin Institute Edinburgh Midlothian EH25 9PS UK; 4 2250 Litton Reaves Hall Blacksburg VA 24061 USA


**Comparative and Functional Genomics**
*Comp Funct Genom* 2005; **6**: 311–316.
Published online in Wiley InterScience (www.interscience.wiley.com). **DOI:** 10.1002/cfg.485

**Meeting Review**

### Poultry genomics puts meat on the table

### Chicken Genomics and Development Workshop, Cold Spring Harbor Laboratory, Cold Spring Harbor, NY, USA, 8–11 May 2005

Ed Smith*[1], Olivier Pourquié[2] and Dave Burt[3]
[1]*Department of Animal and Poultry Science, Virginia Tech, Blacksburg, VA 24060, USA*
[2]*Stowers Institute for Medical Research, 1000 East 50th Street, Kansas City, MO 64110, USA*
[3]*Department of Genomics and Genetics, Roslin Institute, Edinburgh, Midlothian EH25 9PS, UK*

**Correspondence to:*
*Ed Smith, 2250 Litton Reaves Hall, Blacksburg, VA 24061, USA.*
*E-mail: esmith@vt.edu*

Received: 12 June 2005
Revised: 15 June 2005
Accepted: 16 June 2005

### Introduction

Why did the chicken cross the road, you ask? Because the draft sequence of its genome has been released, silly. So, along with other 'bird enthusiasts' and advocates, those long involved in chicken genetics and genetic studies of birds came to **Cold Spring Harbor Laboratory (CSHL), 8–11 May 2005,** for the 3rd Chicken Genomics Workshop to pat each other on the back for the recently released draft sequence (International Chicken Genome Sequencing Consortium, 2004), compare notes, review progress, and plan for the road ahead! Never mind that the meeting preceded, perhaps as a convenience or, to a cynic, just 'being chicken' (an inability to 'stand alone'?), the annual 'Biology of Genomes' meeting: these scientists did not shy away from the 'chicken jokes' or from making a strong case for why their work ranks up there with other tractable biomedical models. Joking aside, many speakers at the chicken meeting also attended or were part of 'Biology of Genomes', so the chicken is now clearly recognized as a model genome and of great value in evolutionary comparisons.

Scientific presentations included the usual staples at genome meetings, such as SNPs, sequence to function, QTL identification and expression profiling. They also included novel talks about gynandromorphs, endogenous viral elements, transgenesis, developmental mutants and signalling pathways. The gathering represented a culmination and a celebration of a vision that started with the partnership between **Jerry Dodgson**, Michigan State, and **Lyman Crittenden**, USDA–ARS, as well as the efforts of several European scientists, including the late **Nat Bumstead, Martien Groenen** (Wageningen) and **Dave Burt** (Roslin Institute). It was appropriate that the meeting ended with an exploration by the chicken community of 'what now?'

### Bioinformatics tools and the chicken

The meeting began with a keynote presentation by CSHL's resident bioinformatics leader, **Lincoln Stein,** who described his most recent foray

Copyright © 2005 John Wiley & Sons, Ltd.

into using computational tools to make the lives and endeavours of biologists a lot easier — the 'Generic Model Organism Database (GMOD)'. As a resource, such a database permits the integration of information on various species and organisms, and is essential if we are to embrace 'systems biology'. The next morning, **Ewan Birney** added to this discourse by talking about ENSEMBL and the emerging tools at EBI that are being put to use in mining useful information from the chicken sequence. Several speakers, including David Torrents from EMBL and Simon Hubbard from Manchester, reported results from applications of these and other tools to comparative analyses of the chicken proteome and genome.

**Lincoln Stein** did not have a chicken joke in the form of a question, but tried to answer in his talk whether the 'chicken needs a model organisms database (MOD)' that can be used to capture information not currently within existing databases. While currently available databases capture sequences and sequence-based data (ESTs, SNPs, etc.) very well, they do not now integrate data that are more chicken-specific (e.g. strain formation, breeding history and QTL). It is Lincoln's hope that MODs will provide a resource for storing, retrieving and manipulating diverse biological data. An example is 'WormBase' (**http://www.wormbase.org/**), a MOD that the *C. elegans* community use to communicate with each other and to link each other with biological resources. His argument, and a persuasive one, is that MODs are a rational progression in a development that will eventually climb to higher and higher levels that will include 'clade databases' and 'systems biology databases'.

Before making the case for ENSEMBL and its value to the chicken genome jockeys, **Ewan Birney** flattered the faithful by indicating that their favorite organism was also one of his, especially for 'having no or few pseudogenes, a neutral rate of 1.5 substitutions per base, and (alas, some support of that human favourite, "you are chicken") good conserved synteny to human'. The chicken DNA sequence has also provided a useful evolutionary comparison for ENSEMBL in carrying out its main task of genome annotation and predicting genes in databases. ENSEMBL's current prediction is that alternative splicing occurs at a significantly higher rate in the chicken genome than in other species. This high level may, according to Ewan, be a result of the incomplete chicken sequence or due to the current use of cDNA for gene prediction and not ESTs in humans and other species. His cautionary note that 'sensitivity is lost' with chicken–human alignments did not dampen his enthusiasm or that of the faithful in the audience for the chicken genome as a central player in the new biology of comparative genomics, even for human-centric projects such as ENCODE. Interestingly, other speakers illustrated that these comparisons work particularly well for developmental genes — one of the chick's strong points.

In his talk about comparative analysis of the chicken proteome, **David Torrents** (EMBL) presented a sketch of where the chicken fits and its importance in the grand scheme of comparative biology from chimp to mosquito in their search for proteins associated with morphological and physiological differences. Their 'blind approach' primarily focused on comparing the chicken proteome to that of different organisms and correlating the differences in domains and orthologous groups with morphological and physiological differences. In the former, 80 000 protein domains were mapped and used as a starting point for comparative analyses of different proteomes. Examples of under- or over-represented domains in the chicken were reported. At another level, orthologous comparisons within the chicken proteome and among the different vertebrates identified proteins conserved or specific in the different organisms. While these approaches are still limited by the incomplete nature of the chicken genomic sequence (is the glass half-full or half-empty?), examples provided from the work of **David Torrents** and his colleagues seem to suggest that, by comparison to human and *Fugu*, the duplication rate or survival of duplicated proteins has been lower in avian genome evolution. One dramatic difference described between the chicken proteome and those of human and *Fugu* was in the large number of sequences similar to feather keratin.

It was fitting that a veteran member of the chicken genome community, **Martien Groenen (Wageningen)**, had something to say about the use of computational resources to mine useful information from the chicken genome. He was appropriately introduced by Jerry Dodgson, as one of

Copyright © 2005 John Wiley & Sons, Ltd. 

the few who could speak in all the sessions at the meeting, having made significant contributions in all areas. Using *Protein World*, a database of 145 proteomes from sequenced genomes, Martien and his colleagues used the ENSEMBL set of 28 000 predicted chicken peptides to conduct 34 million pairwise comparisons with other proteomes, including human, mouse and rat. These comparisons are made to increase the likelihood of more reliably identifying true orthologues and accurate intron–exon boundaries, and to identify proteins involved in protein–protein interactions. The depth of the comparisons indicate that the chicken community will require such collaborations if it is to have access to the large computational resources needed to answer important questions.

### Sequence to function: sex chromosomes

As expected, a significantly higher number of talks focused on the biological value of the sequence, one of **Dave Burt's** (Roslin) biases. A few of the presentations and posters included work on the Z and W chromosomes, which remain of strong interest because of the uniqueness of these chromosomes when compared to the sex chromosomes of eutherians. Interest in the sex chromosomes also illustrates the incomplete nature of the present draft sequence, as the Z and W assemblies contain only 30% and 2%, respectively, of the sequence expected to be on these chromosomes, due to their repetitive nature and lower coverage in sequencing libraries. Currently, 48 genes have been identified on the Z and 10 on the W. Eight of the W genes have orthologues on the Z. It is emerging that sex determination in the chicken, and probably in other birds too, is different from that in mammalian systems. It appears that even though there is a bird homologue for Sry, there may be no single 'master' sex-determination gene in birds. A much-talked about presentation was one from **Michael Clinton** (Roslin Institute) involving gynandromorphs and a novel W-chromosome gene(s) that appears to be involved in sex determination in birds. It turned out that in birds, the role of gonads in sex determination is more limited, and cell-autonomous factors appear to also be involved.

The role of the sex chromosomes also formed the basis of the presentation by **Horst Harmeister** (University of Ulm) of his controversial 'brains and balls' hypothesis. The central concept that males, the heterogametic sex, have a higher level of stress and thus a higher rate of mental retardation and infertility, has, however, not been tested in the chicken, where the female is the heterogametic sex. But evidence was provided of differential expression in chicken and human brains of genes involved in reproduction, including gonadal function. It is yet not clear whether the chicken Z chromosome genes are as conserved as those of the human X and thus what their role may be in the development of new traits.

Other talks about work involving the W were given by **Richard Crooijmans** (Wageningen) and **Sofia Berlin** (Uppsala University). Richard described Z and W mapping results that involved the radiation hybrid panel described at the meeting and also published elsewhere by **Alan Vignal** (INRA) and his colleagues. The sex chromosome-specific radiation hybrids are expected to accelerate the completion of sequence assignment to the very, highly repetitive W. In her presentation, **Sofia Berlin** described her work in **Hans Ellegren's** lab that involved phylogenetic analysis of the sequence features of the chicken W to determine the effect of the lack of recombination. Their data suggest that the chicken W sequences have three characteristics that can be directly attributed to the lack of recombination: accumulation of deleterious mutations; extremely low nucleotide diversity; and degeneration that is reflected in the highly repetitive nature of the chromosome. **Pete Kaiser** (IAH-Compton) finished off with a *tour-de-force* on how he has hunted and mined the genome for chicken immune genes. His work illustrated the power and the deficiencies of the genome data and the need to complete the sequence so we can find genes like those involved in immune response that evolve at such a high rate and be sure they are not missing from birds.

### Sequence to function: genetic resources and genomic reagents

Several groups described genomic reagents, including ESTs, cDNA sequences and SNPs, that appear

Copyright © 2005 John Wiley & Sons, Ltd.

to add to the initial analyses that followed the release of the draft sequence (International Chicken Genome Sequencing Consortium, 2004). For example, **Gane Ka-Shu Wong** reported that, unlike results in humans, at first glance SNP distribution in chickens appears to be uniform and independent of recombination rate. On closer examination, however, there appears to be an association between SNP frequency and recombination rate locally in macrochromosomes. Other noteworthy observations include results, albeit early, that single gene mutations that cause human diseases do not appear to be conserved in orthologous chicken genes. Only one instance was found in 1000 comparisons made of genes in the OMIM database. Gane's talk and his data seem to suggest a lack of selective sweeps in chickens, at least on the order of contiguous 100 kb segments, but we will have to wait for results from population studies using SNP panels of 5000–10 000 markers, planned in the next 12–24 months.

In addition to the SNPs, others also described genetic resources that could be useful for SNP discovery, haplotype analysis and QTL identification. **Mary Delany**, University of California at Davis, also raised the quest, under her leadership, to find a permanent solution to the continued loss of avian genetic resources by providing an update regarding financial resources and the availability of stocks for research. As if to underscore the value of these genetic resources, **Leif Andersson** (Swedish University of Agricultural Sciences), described the Swedish effort to develop a panel of 10 000 SNPs using lines divergently selected for body weight for over 45 generations, among other diverse lines. Complete with dramatic pictures of examples of low- and high-line birds reminiscent of the growth hormone transgenic and non-transgenic mice, the value of both the populations and the SNPs were evident in defining the role of specific genes in causing variation in complex traits that include body weight. These resources, as well as new long oligonucleotide microarrays and Affymetrix chips described by **Richard Talbot** (Roslin Institute; **www.ark-genomics.org**), should keep the chicken genomics community busy for a while as they strive to identify QTLs and become 'sequence functionators'.

### Sequence to function: comparative genomics

There were several talks that may help the community in the long-term quest to answer the important question, really the reason why NIH funded the chicken genome sequencing project in the first place and thus the involvement of the WU Genome Center: 'What is the biological value of the chicken genome sequence, especially to human genetics?'. Consistent with the initial analysis reported by the International Chicken Genome Sequencing Consortium (2004), the emerging additional analyses seem to suggest that less than 3% of the human genome aligns with the chicken, compared to 45% of the mouse. While this results in greater specificity, a significant fraction of functional information within the human genome appears to be missed in comparison with that of the chicken. Evidence presented by **Ross Hardison** (Penn State), suggests that included in the 3% of the human genome that aligns with the chicken genome are ultra-conserved elements which correspond to exon-poor regions or 'stable gene deserts'. The current working hypothesis is that the 'gene deserts' have regulatory elements. **Elliot Margulies** provided another take on comparative genome analyses from the National Human Genome Research Institute. It turns out that the 3% alignment between chicken and human is doubled to 6% if the pairwise comparison is based on a multiple alignment involving multiple species, especially one including non-eutherians. The key point here is that we expect that all these sequences are under selection and therefore of functional value to both chicken and humans. This contrasts with 'noise' found in the comparisons between mammalian sequences. So Elliot's results extend the usefulness of the chicken comparisons — so yes, the sequence of the chicken genome has been and continues to be of great value.

### Sequence to function: developmental biology

Finally, we had an intense session on developmental biology, for which the chick excels. **Parker Antin** (Arizona) and **Richard Buckland** (Edinburgh) talked about current efforts

Copyright © 2005 John Wiley & Sons, Ltd. 

for the collection and presentation of *in situ* hybridization data on gene expression patterns in whole embryos. Parker currently maintains *GEISHA* (**http://geisha.biosci.arizona.edu/**), a repository for gene expression data during early development. Whilst there is a need for such a high-throughput approach, Richard discussed a more detailed system modelled on EMAP, the mouse expression atlas (**http://genex.hgu.mrc.ac.uk/**). This was followed by a number of presentations using the genome sequence to predict regulatory sequences and the chick to test these *in vivo*. **Hiroto Kondoh** (Japan) gave a fascinating presentation on mapping the enhancers that control the pattern of expression of the chicken SOX2 during early chick development. This exploited the genome sequence to predict regulatory sequences by comparisons with human genes and then to directly test these predictions *in vivo* by electroporation of chick neural tubes with a number of GFP fusions. Using similar techniques, **Rob Krumlauf** (Stowers Institute for Medical Research) was able to dissect out regulatory sequences used in HOX gene expression — these were stories the bioinformaticians in the audience marvelled at. The advantages of the chick in developmental studies were further demonstrated by **Claudio Stern** (UCL, London), where using new imaging tools he was able to map out complex signalling pathways during neural development and to follow the behaviour of cells *in vivo*. After the banquet on the final night (!), **James Briscoe** (NIMR-London) entertained everyone with a fascinating story of the complex signalling systems in the chick, including the involvement of sonic hedgehog in determining the pattern of neuronal derivatives in the developing neural tube. **Dave Burt** (Roslin), having delayed his talk until the next morning, proceeded to wake everyone up with his work on positional cloning of the talpid3 mutant, with the discovery of a new member of the SHH signalling pathway — demonstrating that genetics still holds many surprises.

Yasuhiro Kawakami (Salk Institute, San Diego) then presented elegant studies in chick embryo analyzing the left-right patterning system, which is responsible for the asymetrical positioning of our internal organs. A large part of the following session including talks from Olivier Pourquie (SIMR, Kansas City), Christophe Marcelle (IBDM, Marseille) and Andrea Munsterberg (University of East Anglia, Norwich) who discussed the use of the chick embryo to study somitogenesis. Elegant strategies for in vivo imaging in the embryo were presented. Rob Etches (Origen) and Helen Sang (Roslin) presented novel strategies based on primordial germ cells lines or lentiviruses to generate transgenic chicken. The session was concluded by an impressive overview of the complexity of feather development in chicken by Chen-Min Chuong (USC, Los Angeles). Wes Warren from the WashU genome center who sequenced the chicken genome gave the final lecture emphasizing the goals for the near future. Whereas a new release of the sequence will be issued in the summer of 2005, completing the chicken genome sequence remains a major objective.

### Summary

If the faithful had any concerns that their ‘model’ has not added a lot of value to the information being mined from the human genome, it was not evident at the meeting’s end. Rather, I sensed hope and optimism and a clear plan as to what should come next. On the ‘to do’ list are completion of the genome sequence (in particular the sex chromosomes), creation of a chick atlas of development and a MOD, as well as other subjects. The plan is to hold a CSHL meeting every 2 years (keep an eye on the AvianNet www site for news: **www.chicken-genome.org** or CSHL: **http://meetings.cshl.edu/meetings/chick05.shtml** for the next meeting on 7–10 May 2006) to focus on Genome Biology, and to alternate this with a meeting at another location outside of the USA to focus on the Biology of Birds. Claudio Stern (**c.stern@ucl.ac.uk**) will host such a meeting in 2007 in Barcelona, Spain, with a major focus on Development, the Immune System and Evolutionary Biology. Dave Burt also suggested that we will search for support of graduate students and post-docs to participate in future meetings (so any sponsors interested let him know). Even as concerns remain about losing genetic stocks that helped the poultry genetics community make significant contributions to vertebrate biology, participants felt that there was strong interest in the continued use of the chicken in comparative biology. It was clear that the draft of the chicken

Copyright © 2005 John Wiley & Sons, Ltd.

sequence is definitely just 'the end of the beginning', if that.

### Acknowledgement

We are grateful to Jerry Dodgson, Michigan State University, for his input as well as for his editorial comments and suggestions on the manuscript.

### Reference

International Chicken Genome Sequencing Consortium. 2004. Sequence and comparative analysis of the chicken genome provide unique perspectives on vertebrate evolution. *Nature* **432**: 695–716.

Copyright © 2005 John Wiley & Sons, Ltd. 
